# Human–robot interaction for social skill development in children with ASD: A literature review

**DOI:** 10.1049/htl2.12013

**Published:** 2021-05-19

**Authors:** Anastasia Raptopoulou, Antonios Komnidis, Panagiotis D. Bamidis, Alexandros Astaras

**Affiliations:** ^1^ Lab of Medical Physics School of Medicine Aristotle University of Thessaloniki Thessaloniki 54124 Greece; ^2^ Computer Science American College of Thessaloniki Thessaloniki Greece

## Abstract

Human–robot interaction has been demonstrated to be a promising methodology for developing socio‐communicational skills of children and adolescents with autism spectrum disorder (ASD). This paper systematically reviews studies that report experimental results on this topic published in scientific journals between the years 2010 and2018. A total of 1805 articles from various literature were filtered based on relevance and transparency. In the first set of criteria, article titles are screened and in the second both titles and abstracts. The final number of articles which were subsequently thoroughly reviewed was 32 (*N* = 32). The findings suggest that there are benefits in using human–robot interaction to assist with the development of social skills for children with ASD. Specifically, it was found that the majority of studies used humanoid robots, 64% relied on a small number of participants and sessions, while few of the studies included a control group or follow‐up sessions. Based on these findings, this paper tried to identify areas that have not been extensively addressed to propose several directions for future improvements for studies in this field, such as control groups with typical developmental children, minimum number of sessions and participants, as well as standardization of criteria for assessing the level of functionality for ASD children.

## 1 INTRODUCTION

An increasing number of people across the globe are being diagnosed with autism spectrum disorder (ASD). In 2018, the CDC estimated that the average prevalence is one out of 59 children, diagnosed with ASD. It has been observed that the ratio of boys to girls with autism is 4:1, so the chances of being born a boy with autism are much higher [1]. However, recognition of autism in girls is a challenge not only due to sex‐based diversity, but also because most diagnostic tools have been developed primarily based on the observation of boys' behaviour [2]. This results in the possibility of ignoring cases of autistic girls who simply do not show behaviour observed in boys. Without the diagnosis of these girls, the proportion of boys with autism maybe increases.

Nowadays, according to the revised version of the diagnostic criteria published as part of DSM‐V, children with ASD encompass a wide variety of symptoms but are generally characterized by difficulties in two different behavioural aspects: Social communication, stereotypical, repetitive behaviours or activities associated with unusual sensory stimulation.

Possibly the most important challenges for individuals with ASD are social interaction and their socio‐emotional development in general [3]. This difficulty in social interactions is the result of impaired linguistic and communication skills, often combined with deficiencies in cognitive skills [4]. Individuals with neurotypical development possess communication skills based on their inherent capacities for social interaction. However, it is difficult to focus on the development of communication isolated from sociability [5]. This term sociability refers to a person's ability to adapt to social conditions and to engage in friendly relationships and activities.

For individuals with autism, communication and speech disorders are often intertwined, giving a wrong impression about their profile, as the concept of communication disorders concerns the development of sociability and interaction prior to the development of speech [6]. Speech disorders relate to the difficulties of developing language when the person, while having the desire to interact and communicate with others, does not know how to implement it. Therefore, it appears that for many individuals, autism is a cognitive disorder that affects their development of social and communication skills. However, the disorder of speech or language is not the primary difficulty of individuals with autism, as language is a tool and a means of communication. Therefore, the difficulties in communication are those that cause problems in the development of the language, as the person does not really know how to use it [6].

Children with ASD often have the desire to develop social relationships and make friends but find it difficult to create and maintain friendships, as they do not understand basic behavioural rules. This may lead to feelings of anxiety with respect to social interactions, which sometimes leads to socially unacceptable behaviour [7]. They may also find it difficult to interact smoothly with their peers in social games, which consequently limits their ability and opportunities to apply social strategies and gain credibility in social or friendly relationships [8, 9, 10].

A potentially important direction for research in ASD is the identification and development of technological tools which can make the application of intensive treatment more readily accessible, effective and cost‐effective. In response to this need, a growing number of studies have been investigating the application of advanced interactive technologies to address core deficiencies related to autism, such as in social communication. Therefore, there is an urgent need to recognize the necessity of providing treatment and education to those people.

In recent decades, educational robotics has been used to develop and educate social skills in children with ASD. Several surveys [11, 12] report encouraging results obtained from attempts to implement educational robotic programs in children aged between 4 and 18 years old: increased levels of attention, improved social skills, imitation learning and active participation of children through their interaction with the robot. Further research findings have shown that anthropomorphic robots may have greater potential in ASD therapy with respect to skills generalization [13], because they engage and maintain the children's interest during therapy sessions [14].

Balancing the level of realism of anthropomorphic robots used in ASD therapy is crucial. If the humanoid looks too human‐like the child may develop fear and/or lack of interest. On the other hand, it should not look too machine‐like because the child will be more interested to examine it rather than interact with it [15]. Humanoids adopting a cute, attractive design (i.e. with big eyes, curved shape, highly expressive body language and facial expressions) contribute to rich gaze expressions and help avoid fearfulness among children with ASD [16]. They have also been reported to be more approachable by children with ASD [13]. This review paper investigates how human–robot interaction is being used to improve the social skills of children with ASD.

## 2 MATERIALS AND METHODS

The articles surveyed for this review were obtained through bibliographic research in some of the major peer‐reviewed scientific literature databases such as PubMed, ScienceDirect and SpringerLink, as well as Google Scholar. The initial keywords used were autism and robot in various forms.  For instance, compound Boolean search statements were used, such as the following one focusing on the popular robotic kit Lego Mindstorms: ((autism OR autistic OR ASD OR developmental disorders OR Asperger) AND (robot OR robotic OR robotics OR Lego OR NXT OR EV3 OR Mindstorms)).

The search was set to the plain text of the publication results. The papers were limited to a publication date between the years 2010 and 2018, in peer‐reviewed scientific journal format and written in English. Conference proceedings as well as books or book chapters and papers published prior to 2010 were excluded from the search results. For this purpose, appropriate filters were implemented in each of the search engines utilized. The year 2010 was chosen as our straining limit because we thought that in a rapidly evolving technological area such as robotics, papers older than 7–8 years could refer to technology considered partially obsolete.

A total of 1805 articles were selected between the years 2010 and 2018. Moreover, to further refine these search results, a number of two exclusive criteria were applied. As the first set of refining criteria, we screened the titles of the articles and assigned:

- Οne (1) point if there are both “autism” and “robot” (including variations) were present anywhere in the article title.
- One (1) point if the article was published in a peer‐reviewed scientific journal.
- One (1) point if the article was written in English.

For an article to pass this first set of selection criteria, it had to accumulate all three (3) points.

In the second set of selection criteria, we screened both the titles and abstracts of the articles and assigned a number of qualification points as shown below:

- Two (2) points if there was a clear reference to social skills.
- Two (2) points if the number of study participants was clearly mentioned in the article (provided experimental research was conducted, otherwise no points were awarded).
- One (1) point if the participants of the published research were children and/or adolescents.
- One (1) point if the name of the robot used in the published research was clearly mentioned in the article.

**FIGURE 1:**
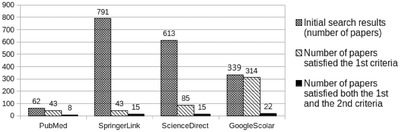
The number of the articles remained after the application of the exclusive criteria

For an article to pass this second set of selection criteria, it had to accumulate at least five (5) points. An analysis of the articles remaining after applying both sets of selection criteria is depicted in Figure 1.

The total number of articles remaining at this stage was 60 (*N* = 60). After the withdrawal of duplicates across different databases, as well as the removal of MSc and PhD thesis dissertations, this number was further reduced. The final number of remaining articles which were subsequently reviewed was 32 (*N* = 32) [15, 18–48]. The majority of articles obtained through this process were published in the year 2018 (nine out of 32), as the development of technology in education combined with educational robotics has created new research data in recent years. The other articles were distributed among years ranging between the year 2010 and 2018.

With respect to the first set of screening criteria for articles, it was important to mention the name of the robot that was used by the researchers. A variety of robots were employed in different interventions to help children with ASD improve their social and communication skills. The most commonly used robots were:

- **Nao (**Aldebaran Softbank Robotics, Japan) is an autonomous and programmable humanoid robot. It is equipped with multiple sensors and acquires multiple modalities of data from its environment. It can speak and move its limbs independently, walk, and even dance – using its 25 DOF (degrees of freedom) body (https://www.softbankrobotics.com/emea/en/nao).
- **Lego Mindstorms** (The Lego Group, Denmark) is a programmable robotic kit developed by LEGO. It can be used to build different robotic designs equipped with a variety of sensors and motors, all connected to a main “brick” which houses the programmable microcontroller and battery. The most powerful feature of this kit is its simple programming environment, which can be used by children of young age (https://www.lego.com/en‐us/mindstorms).
- **Probo** (Vrije Universiteit Brussel) is a research prototype animal‐like robot that can offer a unique (if slightly eccentric) human–robot interaction. It features a moving head and eyes which can track people around it, while it can also move its mouth, ears and trunk to emulate emotions using body language (http://probo.vub.ac.be/Probo/).
- **Kaspar** (University of Hertfordshire) is a child‐sized humanoid robot capable of a range of simplified facial expressions. It senses and responds to the touch of children and can move its arms, head and eyes (http://assistive‐technology‐for‐autism.wikia.com/wiki/Kaspar).

Moreover, some remarkable other robots worth mentioning for various reasons were:

- The robotic toy **Keepon** (Hideki Kozima, Japan; BeatBots LLC, USA) is a platform which has been widely used in humanoid interaction studies involving social behaviours. Keepon has the shape of a yellow snowman and is equipped with two cameras and a microphone in the place of its eyes and nose, respectively. Its body, which is made of soft silicone and deforms when someone touches or squeezes it,  is capable of limited movement caused by four DC motors. It stands out due to the affective qualities despite its relative technological simplicity (https://en.wikipedia.org/wiki/Keepon).
- **Pekoppa** (Sega Games Co, Japan) is a battery‐powered plastic robotic plant, manufactured which reacts with nodding movements when being spoken to. This robot stands out due to the bold attempt to bestow effective qualities to a plant‐shaped robot (https://en.wikipedia.org/wiki/Pekoppa)

The list above does not include robots that were only used in a single study covered in this paper, unless it was a particularly unusual or uniquely capable research prototype.

## 3 RESULTS

**FIGURE 2:**
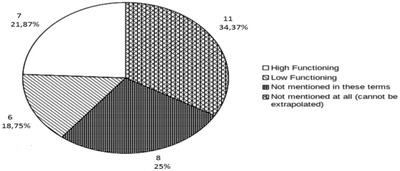
The level of functionality of the children with ASD participated in the research articles we investigated

These articles were analyzed with the help of a questionnaire specifically made for this purpose (https://goo.gl/forms/LUrn9Qfi4iHvR29E2). Specifically, in this systematic review twelve (12) different variables were studied, based on the questionnaire we created for the purpose of grouping, studying and separating the articles we identified. Three variables were used which relate to journals: the title of the scientific research project, the year of publishing and the type of publication. The other four variables related to participants in each study. First, we focus on the number of participants in each study, especially the number of children with ASD in contrast to children of neurologically typical‐developing children, where it existed control group.  Second, we group children participants according to age. Third, we screen for the type of sample, because in some research studies the participants are both children and teachers, even parents. Finally, we categorize the children with ASD according to their functionality (Figure 2). It is important to mention that this variable was a somewhat difficult to screen, because the writers of the articles use different ways to explain the functionality of the children with ASD. Only a few of them used the DSM‐V criteria. In conclusion, these variables were particularly useful for us to export conclusions.

The other five variables are related to research data and especially to the number of sessions, the names of the robots that they are used, the type of the robots and robotic setup and finally the type of measurement that is used.

Specifically, the number of the session are grouped between three groups, one to four sessions (1 to 4 sessions), five to ten sessions (5–10 sessions) and more than ten sessions (>10 sessions). The results show that 16 articles (5%) have one to four session interventions, in contrast to 10 articles (31%) which have more than 10 sessions, while only six articles have five to ten sessions (19%). The majority of researchers used a small number of sessions and maybe this happened due to the type of sample and the difficulties that the researchers faced with.

On the other hand, the questionnaire separates the robots to non‐humanoid, animal or plant‐like and humanoid. More analytically two (*N* = 2) research projects used non‐humanoid robots, five (*N* = 5) animal or plant‐like robots and the majority of studies (*N* = 25) used humanoids, while 17 studies specifically used the anthropomorphic robot Nao (53% of the overall articles).

More specifically about the robotic experimental setup, eighteen (*N* = 20) research studies used commercially available as a pre‐assembled robot, two of them (*N* = 2) commercially available robotic kits, eight (*N* = 8) of the robots were custom‐designed and built by the researchers and finally, two (*N* = 2) of them are customized robots based on a commercially available model.

Finally, it is important to mention that the majority of the articles (56%) did not use a control group of typically developing children, while only 44% of the researchers used children both with and without ASD. However, it is worth mentioning that in several articles a different type of control group was used. The research presented in this review paper focuses only on articles which possess a control group comprising typically developing children, since that would be particularly valuable for planning future research. Moreover, different types of measurements were identified for data acquisition, such as observation, questionnaires, interview, video and others. The most flexible – and therefore promising – of these modalities appeared to be video footage, since it enables researchers to repetitively observe the effects of their interventions on children's behaviour.

According to ICF‐CY (The International Classification of Functioning, Disability and Health for Children and Youth), we create screening criteria to categorize the interventions of each article according to communication and social skills. Specifically, the categories of communication skills we used were: to learn a new form of communication; to make contact with other children (not necessarily to initiate it); orientation to listen (and follow verbal instructions); talk and use verbal abilities; understand the intention of gestures; understand intentions described by an image; understand subtlety of intentions of words; use gestures and non‐verbal abilities.

With respect to social skills the categories we identified and used based on the ICF‐CY were: imitation; attention; appropriate coping with one's anger /sadness; awareness of one's feelings, wishes, behaviour; appropriate reaction to the behaviour of others; social routines (greet, say goodbye, introduce); turn‐taking behaviour; respect/value others (or things); appropriate behaviour with respect to physical, proximity/contact or personal space; collaboration / joint attention; request help; conflict management; social/interpersonal interactions and relations.

More specifically in the area of communication skills, the majority of articles investigated abilities related to verbal communication (*N* = 9) and whether the children with ASD could make contact amongst themselves or with children of neurotypical development (*N* = 11). It is important to mention that most of the projects researched a combination of communication and social skills. Social skills of particular interest to researchers appeared to be the children's attention span (*N* = 17), their ability to collaborate with others (*N* = 13) and imitation (*N* = 13). These types of observations may appear more frequently in the scientific literature partly because it is easier to obtain relevant results.

## 4 DISCUSSION

The rapid increase in several studies investigating the use of robots in improving the social skills of children with ASD in the last decade is remarkable. This indicates a belief within the robotics and social sciences communities that robots have a promising, beneficial impact on special education. By studying the publications released over 2010–2018 time period, we have reached some conclusions and can offer certain suggestions:

1. The first aspect that quickly stood out is that a clear majority of published research covered in this review used anthropomorphic robots. This was to be expected since, according to special education researchers, anthropomorphic robots are more suitable for children with ASD. Anthropomorphic robots are more reachable; they can stimulate and maintain the interest of the children without causing distraction [14], and they can facilitate the generalization of the skills learned. According to [17] “one of the reasons that people may respond differently to human‐like technologies than to machine‐like technologies is because they feel more similar to the former and thus experience more shared identity with them.”
2. A large portion of the studied publications (about 64%) used a small sample size of children with ASD (<12 subjects) and 17% of the studies used a sample of merely 1–4 subjects. These sample sizes can only indicate a trend and are far from establishing scientific certainty. Of course, none of the researchers claimed otherwise, instead they all suggested the pursuit of further studies with larger sample sizes.
3. 56% of the investigations studied included no control group of typically developing students in their experimental protocol. This is probably because it is difficult to set up two almost identical groups (the experimental group and the control group), especially when it comes to finding subjects with special education needs: ASD refers to a wide spectrum of disorders and therefore a varied mix of abilities and impairments. Therefore, the differences between children with disorders in the autistic spectrum can be large and even the same child at different times may react dramatically different to the same stimulus. Furthermore, biological age usually does not coincide with the cognitive age of the child, with variable differentiation among individuals. All this makes it difficult to adopt reliable and objective weighted criteria for comparison and assessment among children with ASD to create equivalence between groups.

Matters become even more complicated as there is no common methodology in assessing and describing the social functionality level of children described across different studies and scientific publications. Some researchers are using the terms “high/low functionality”, others introduce a third “intermediate” state, some rely on IQ scores, while others use a plethora of weighted tests for children's individual skills such as oral communication and/or other social skills.

Instead of using a control group of children without ASD, some researchers attempted to establish a different type of control group using the A‐B‐A‐B schema, where A represents the baseline and B represents the intervention. In this schema, multiple baselines between interventions act as a form of comparative measure, in the absence of a control group. While the use of the A‐B‐A‐B schema is common practice in social sciences, the use of a proper control group comprising typically developing children would still have been preferable. In one research paper [18], an A‐B‐A‐C schema was reported: in this case A was considered as the baseline (the child's behaviour in the classroom without any intervention), B was considered to be the human intervention and C was considered to be the robotic‐assisted intervention.

4. With regards to the number of sessions in the research reports we studied, what surprised us was the fact that they oftentimes used either too few (1 to 4) or a rather large number (>12) of sessions per subject, with only a small number of surveys relying on intermediate numbers of sessions. Particularly in the first category, the surveys that used only one session were the majority. This approach may make sense in terms of evaluating acceptance of a robot by the child participant, however, at the same time it excludes information about habituality and sustainability of acceptance, as well as whether any advantages of robotic interaction vs interaction with a human last the test of time.

We propose the adoption of experimental protocols which provide data from a large number of sessions (>12), the first few sessions used for the extraction of information about the acceptance of the robot by the child participant, subsequent sessions used for drawing more holistic conclusions about the effectiveness of the project's scope, while periodic follow‐ups can be used to investigate the sustainability of results.

5. Although there was no provision in our questionnaire for the recording and presentation of follow‐ups, reading all these studies, we deducted that few (<5) of the projects included follow‐up surveys. Moreover, even those studies that did include follow‐ups, they had implemented them following a rather short period (for instance, 1–3 months).

## 5 CONCLUSION

In this review, we searched the major online databases for articles related to children with ASD, social skills and robots. We investigated 1805 articles published between the years 2010 and 2018. After the application of a set of weighted criteria, we ended up with a reduced number of 32 articles. These articles were analyzed with the help of a questionnaire specifically made for this purpose to extract valuable information about the participants, the robotic platform used, the experimental protocol and the targeted social skills.

Some of the findings were to be expected, for instance, the predominance of anthropomorphic robots since the studies involved social skill development. Other findings were more surprising, for instance the prevalent absence of control groups in experimental procedures and/or the absence of follow‐up reports in most of the articles investigated. We judged the majority of reported studies to be rather limited in size, using either a small number of participants, conducted for a short period and/or for a small number of sessions. This may be sufficient to indicate a trend, a preference or acceptance of a particular robotic platform by the subjects, but it is not conclusive in establishing scientific fact.

Researchers involved in the reported studies relied on different weighted tests to describe the functionality and social potential of the children participants with ASD. This lack of homogeneity in the used terms makes the comparative review and evaluation of results from such studies a complicated and cumbersome task.

We therefore suggest:

- Adopting a common methodology for describing and assessing the functionality and social skills of children with ASD, prior to setting up such experimental protocols. This would make future surveys compatible and their results more amenable to comparison and easier to study.
- We do not believe that there is a need for the creation of a new weighted test, as there are already several proven and reliable tests. However, there is a clear need to select and adopt one of them as part of the aforementioned experimental protocol standard, possibly enriching it with pertinent features obtained from other tests.
- A special care should be taken to organize larger studies, the numbers of participants being much larger than *N* = 12.Ensuring the establishment of a control group comprising typically developing children.
- Securing the budget for a sufficient number of sessions for each subject (ideally larger than *N* = 10, certainly not 1 or 2).
- A series of follow‐up sessions both in the immediate (1–3 months after the intervention) as well as the more distant future (>1 year).
